# Hyperferritinemia and Macrophage Activation Syndrome in Septic Shock: Recent Advances with a Pediatric Focus (2020–2025)

**DOI:** 10.3390/children12091193

**Published:** 2025-09-08

**Authors:** Efrossini Briassouli, Natalia Syrimi, Stavroula Ilia

**Affiliations:** 1 Second Department of Paediatrics, “P. & A. Kyriakou” Children’s Hospital, National and Kapodistrian University of Athens, 11527 Athens, Greece; n.syrimi@gmail.com; 2 Pedatric Intensive Care Unit, University Hospital, School of Medicine, University of Crete, 71110 Heraklion, Greece; stavroula.ilia@uoc.gr

**Keywords:** macrophage activation syndrome, sepsis, septic shock, children, hyperferritinemia, hyperinflammatory syndromes, differential diagnosis, review

## Abstract

Macrophage activation syndrome (MAS), a hyperinflammatory condition driven by uncontrolled immune activation, is widely recognized as a critical complication in pediatric septic shock. This syndrome shares pathophysiological features with hemophagocytic lymphohistiocytosis (HLH) and other cytokine storm syndromes, and it contributes to significant morbidity and mortality in pediatric and adult patients. Hyperferritinemia—a hallmark of MAS—is not only a diagnostic clue but also a prognostic marker for poor outcomes in sepsis. High ferritin levels are strongly suggestive of MAS, yet even moderate elevations in combination with the trend of ferritin levels can be indicative of heightened mortality risk. Distinguishing MAS from severe sepsis or other hyperinflammatory syndromes in children (such as multisystem inflammatory syndrome in children (MIS-C)) can be challenging, as clinical features often overlap. However, early recognition and timely immunomodulatory therapy, particularly corticosteroids and targeted biologic agents, can be life-saving. Recent advances emphasize a syndromic approach to diagnosing MAS within the spectrum of hyperferritinemic sepsis, using scoring tools or MAS-specific criteria adapted to sepsis or MIS-C contexts. Ongoing studies aim to refine biomarker-based stratification and therapeutic algorithms. This review synthesizes current knowledge on MAS as a complication of sepsis, including the diagnostic importance of ferritin levels, differential diagnosis with other cytokine storm syndromes, and the latest therapeutic approaches. It underscores the importance of early suspicion and intervention to reverse immune dysregulation and improve outcomes in critically ill pediatric patients.

## 1. Introduction

Sepsis remains a leading cause of mortality in both pediatric and adult intensive care units worldwide [1]. Current treatments focus on eliminating the source of infection and supporting organ failure, while the proceeding inflammatory disease processes and pathobiology are still unclear. In recent years, attention has turned towards a subset of patients who develop an exaggerated immune response marked by hyperinflammation and multi-organ dysfunction [2]. Among these hyperinflammatory syndromes, macrophage activation syndrome (MAS)—a form of secondary hemophagocytic lymphohistiocytosis (HLH)—has emerged as a pivotal driver of disease severity and poor outcomes in septic shock [3]. MAS is characterized by uncontrolled activation of macrophages and T-lymphocytes, resulting in a cytokine storm, hemophagocytosis, and high circulating ferritin levels [4]. Ferritin, in this context, serves as both a biomarker and a pathogenic mediator, and its measurement can aid in both early detection and prognostic stratification of septic patients at risk of MAS [5].

Despite its clinical importance, MAS remains under-recognized in sepsis, partly due to overlapping features with other inflammatory conditions such as severe bacterial infections, MIS-C, or autoimmune flares [4]. Furthermore, diagnostic criteria for MAS and HLH were originally developed in pediatric oncology or rheumatology populations, making them imperfect tools for sepsis-related presentations [6,7]. As our understanding of cytokine storm syndromes expands, there is a growing consensus that MAS in sepsis should be conceptualized as part of a broader spectrum of hyperferritinemic conditions, each requiring timely immunomodulatory therapy [8,9]. This review explores the pathophysiology, diagnostic role of ferritin, differential diagnoses, and therapeutic strategies for MAS in septic contexts, with special attention to recent advances in pediatric clinical practice.

## 2. Pathophysiology of MAS in the Context of Sepsis

**Figure 1:**
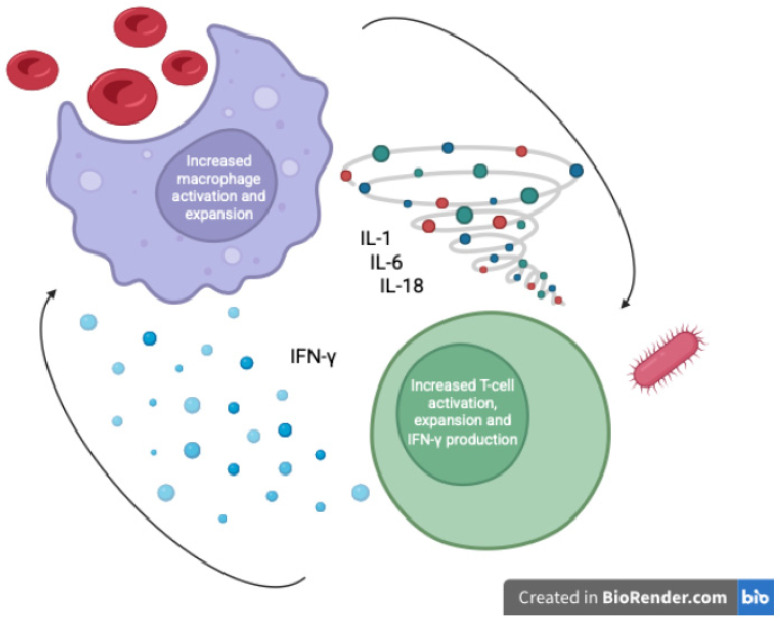
Macrophage activation and hyperferritinemia in pediatric septic shock (author-created schematic).

MAS is an extreme immune response wherein excessive activation of T-lymphocytes and macrophages leads to a cytokine storm and multi-organ damage. In the context of septic shock, a subset of patients develops a “sepsis-associated HLH/MAS” phenotype—sometimes termed macrophage activation-like syndrome (MALS)—characterized by persistent fever, cytopenias, coagulopathy, hepatosplenomegaly, and hyperferritinemia [10]. Phenotypes based on the inflammatory profile of children with sepsis-induced multiple organ failure have been described, where MALS represents the final common pathway of uncontrolled inflammation [11]. The driving immunopathology involves uncontrolled cytokine release (e.g., IL-1β, IL-6, IL-18, IFN-γ) and impaired cytotoxic cell function, resulting in the accumulation of activated macrophages that perform hemophagocytosis (engulfing blood cells in bone marrow and spleen) [12] (Figure 1). These macrophages release massive amounts of ferritin—an acute phase reactant—under the influence of inflammatory cytokines such as TNF, IL-1, and IL-6, which upregulate ferritin synthesis via NF-κB pathways [13]. Autopsy studies of adults who died of sepsis have directly linked hemophagocytic macrophage infiltration in organs to the pattern of multi-organ failure (particularly hepatic dysfunction and disseminated intravascular coagulation (DIC)) seen in fatal septic shock [12]. Notably, such patients had exceedingly high circulating levels of IL-1, IL-6, IL-8, and IL-10, reflecting a cytokine storm indistinguishable from classical MAS [12]. Thus, in septic shock (both pediatric and adult), hyperferritinemia can be a biomarker of a MAS-like hyperinflammatory state driving organ failure. This hyperinflammatory syndrome is often referred to collectively as a “Cytokine Storm Syndrome” (CSS), a category that includes MAS, secondary HLH, and other hypercytokinemic conditions [10]. Importantly, the syndrome feeds on itself: high cytokine levels both result from and further activate macrophages and lymphocytes, unless interrupted by therapy. The end result, if untreated, is progressive multiple organ dysfunction, immune paralysis, and death in a significant fraction of cases [3,13].

## 3. Diagnostic Role and Prognostic Value of Serum Ferritin

Serum ferritin is a central diagnostic and prognostic marker in HLH/MAS and is often the first red flag for hyperinflammation in a septic patient. Marked hyperferritinemia is included as a criterion in all HLH/MAS diagnostic frameworks [14] (Table 1). The conventional HLH-2004 criteria were originally developed by the Histiocyte Society and consider ferritin ≥ 500 ng/mL as one of eight diagnostic criteria aiming to detect familial (primary) HLH [6]. In 2016, the Paediatric Rheumatology International Trials Organisation (PRINTO) introduced updated classification criteria for MAS associated with systemic juvenile idiopathic arthritis (sJIA). These criteria use a higher ferritin cutoff (>684 ng/mL) [4,14] and have also been proven helpful in identifying MAS in the context of other pediatric inflammatory conditions such as Kawasaki disease (KD) and juvenile dermatomyositis (JDM) [7]. In 2014, Fardet et al. invented a scoring system (HScore) different from the HLH-2004 criteria by incorporating variables such as aspartate aminotransferase (AST) levels and underlying immunosuppression, while excluding assessments of NK cell function and sCD25 levels. HScore has 93% sensitivity and 86% specificity for diagnosing MAS and has been widely used since [15]. Lately, Avrusin et al. in 2024 elaborated diagnostic criteria allowing for the differentiation of MAS in MIS-C patients with a sensitivity of 100% and a specificity of 94.9% [16,17]. These oncology- and rheumatology-derived tools have important limitations in septic shock, with substantial risk of misclassification in children; sepsis-adapted, pediatric-specific criteria are needed.

In practice, patients with true MAS in sepsis usually manifest far higher levels: ferritin > 1000 ng/mL, and extreme hyperferritinemia (often into the several thousands) is common [4]. Critically ill children presenting with sepsis and MAS manifestations demonstrated markedly elevated ferritin levels (623–28,931 ng/mL) prior to intervention, as documented in a recent study [18]. Pediatric rheumatology data indicate that ferritin levels > 10,000 ng/mL are >90% sensitive and specific for MAS in children, often considered virtually pathognomonic of HLH/MAS [4]. Adult studies similarly note that ferritin value above 3000–5000 ng/mL in the context of septic shock or inflammatory illness is highly concerning for a hyperferritinemic syndrome (MAS/HLH) [12]. Indeed, one adult series coined the term “hyperferritinemic syndrome” for patients with ferritin above 3000 ng/mL presenting with features of MAS (such as those related to adult-onset Still’s disease, catastrophic antiphospholipid syndrome, or septic shock) [12].

Beyond its role in diagnosis, ferritin provides prognostic information in septic shock. Multiple studies over the past two years have reinforced that even moderate elevations (e.g., >500–1000 ng/mL) are associated with worse outcomes in sepsis [12], and recent studies in pediatric populations with severe sepsis set lower cutoffs for ferritin levels (500–705 ng/mL) to predict mortality risk [19,20]. In a multicenter pediatric cohort, children with “hyperferritinemic sepsis” (ferritin > 500 ng/mL) had a fourfold higher odds of mortality than those without hyperferritinemia (23% vs. 5.7% mortality) [12]. Notably, mortality rose stepwise with ferritin strata: children with ferritin ≥ 10,000 had extremely poor outcomes [12]. Similarly, extreme ferritin elevations in adults with septic shock correlate with a high risk of death; one report found the highest ferritin values (often >9000 ng/mL) predominantly in non-survivors [16]. Moreover, Carcillo et al. proposed the use of combination of ferritin and C-reactive protein to assess the risk of mortality and the treatment effect of sepsis in children [11] Apart from a one-time cutoff, ferritin kinetics are important: a sharp rise in ferritin during the course of sepsis can herald impending MAS [12], whereas a decline with therapy often signals response. In the PROVIDE trial, ferritin concentration remained significantly elevated, even after immunomodulatory treatment (anakinra) was discontinued in critically ill adult patients with MALS [17]. Based on this evidence, extended immunomodulatory therapy until normalization of ferritin levels was proposed. Serial ferritin tracking is therefore useful to monitor disease activity and therapeutic response, and a failure of ferritin to fall (or a secondary spike) should prompt escalation of immunomodulatory treatment [4]. That said, clinicians must remember that ferritin is an acute phase reactant and not specific to HLH—it can be elevated in typical bacterial sepsis, liver failure, malignancy, or trauma. For example, one retrospective analysis of 163 hospitalized children with ferritin above the HLH-2004 cutoff found that only ~5% had HLH, and even when ferritin level was above 10,000 ng/mL the positive predictive value for HLH was only 18% [18]. Thus, hyperferritinemia should always prompt inclusion of HLH/MAS in the differential (especially when very high), but diagnosis still requires compatible clinical features and lab constellation [19]. In practice, a threshold like ferritin > 500 or >1000 ng/mL in septic shock should trigger a systematic evaluation for HLH/MAS (e.g., applying an HScore (Table 1) or checking other criteria such as cytopenias, fibrinogen, soluble IL-2 receptor) [4]. Values climbing into the thousands demand urgent consideration of immunosuppressive therapy, even in parallel with infectious workup.

**Table 1:** Diagnostic criteria for MAS/HLH in children and ferritin levels.

Citeria Set	Ferritin (ng/mL)	Other Key Criteria	Diagnosis	Notes
**HLH-2004**	≥500	Cytopenias, splenomegaly, hypertriglyceridemia, hemophagocytosis, low NK cell activity, high sCD25 (sIL-2R)	5 out of 8 key criteria	Not specific for MAS; used in oncology [6]
**MAS-2016 (sJIA)**	>684	Platelets ≤ 181 × 10^9^/L, AST > 48, TG > 156, Fibrinogen ≤ 360	High ferritin + ≥2/4 criteria	Validated in sJIA; bedside criteria [7]
**MIS-C MAS (2024)**	>469	Platelets < 114 × 10^9^/L, splenomegaly, CNS symptoms, hypotension	Ferritin > 469 AND Platelets < 114 × 10^9^/L, Exclude other shock syndromes	Preliminary; tailored to MIS-C context [21]
**HScore** **(Points)**	>2000 ng/mL	Cytopenias, hepatosplenomegaly, fever, hypertriglyceridemia, elevated AST, low fibrinogen, hemophagocytosis, known immunosuppression	Points assigned to features; Sum of points ≥ 169	93% sensitivity and 86% specificity for diagnosing MAS [22]

MAS, macrophage activation syndrome; HLH, hemophagocytic lymphohistiocytosis; HScore, hemophagocytic syndrome diagnostic score; MIS-C, multisystem inflammatory syndrome in children; CNS, central nervous system; AST, aspartate aminotransferase; TG, triglycerides; sCD25 (sIL-2R), soluble interleukin-2 receptor; NK, natural killer.

## 4. Differentiating MAS, HLH, and Other Cytokine Storm Syndromes

MAS and secondary HLH are overlapping entities—some experts use the terms interchangeably when referring to hyperinflammatory syndromes. Technically, HLH is the broad syndrome of hemophagocytic lymphohistiocytosis, which can be familial (primary) due to genetic mutations or secondary to triggers like infection, malignancy, or autoimmune disease [4]. MAS is often used to describe HLH arising in the context of rheumatologic disease (classically systemic JIA in children or adult-onset Still’s disease), but mechanistically it is a form of secondary HLH [23]. In septic shock, the hyperinflammatory state has been called “sepsis-associated HLH” by hematologists and simultaneously labeled “MAS” by rheumatologists—underscoring that these syndromes lie on the same spectrum [24]. Indeed, a severe case of hyperferritinemic septic shock with DIC, hepatobiliary dysfunction, cytopenias, and high fevers might meet diagnostic criteria for HLH and MAS simultaneously, even though historically HLH criteria were designed for pediatric oncology settings and MAS criteria for rheumatic disease flares. Differentiating an HLH/MAS flare from “just severe sepsis” can be challenging; they often mimic each other, and in fact may coexist. Key clues favoring HLH/MAS include disproportionately high ferritin levels, trilineage cytopenias not explained by dilution or DIC alone, hypofibrinogenemia (fibrinogen consumption from hemophagocytosis), and evidence of immune dysregulation such as low NK cell activity or very high soluble IL-2 receptor (sCD25) [10]. In children, the presentation of primary viral HLH or MAS can initially appear like severe septic shock with multi-organ dysfunction syndrome (MODS), but such patients often fail to improve with standard antiviral/supportive care. A useful bedside prompt from recent guidelines is to remember the “three Fs” when standard therapies are not working: fever, falling blood counts, and high ferritin [23]. The presence of all three should raise suspicion for HLH/MAS in a deteriorating patient, either pediatric or adult, and should expedite consultation with hematology/rheumatology and initiation of HLH-directed workup.

It is also important to distinguish MAS/HLH from other cytokine storm syndromes that share features but have different contexts. For instance, cytokine release syndrome (CRS) in CAR-T cell therapy or severe COVID-19 can cause fever, ferritin elevation, and organ dysfunction, but the management might differ (tocilizumab is first-line in CAR-T CRS, whereas HLH requires broader immunosuppression). Likewise, Kawasaki disease or toxic shock syndrome in children can present with hyperinflammation and even hemophagocytosis in some cases [14], yet these conditions are not primarily disorders of immune cell cytotoxicity. Overlap is frequent—for example, a child with severe Kawasaki disease or Epstein–Barr virus infection can demonstrate secondary HLH, and conversely, a patient with genetic HLH may be tipped into a flare by a common infection. Thus, clinicians must maintain a high index of suspicion and often pursue parallel diagnoses, e.g., treating presumed sepsis and investigating HLH simultaneously.

Modern scoring systems like the HScore (which assigns points for fever, organomegaly, cytopenias, ferritin level, triglycerides, fibrinogen, etc.) are used in adults to estimate the probability of HLH in critically ill patients [3,12]. In children, updated classification criteria (MAS-2016 for sJIA, etc.) help identify MAS earlier, and they intentionally exclude findings like hemophagocytosis (which may or may not be present on bone marrow biopsy) because waiting for a marrow aspirate can delay life-saving treatment [4]. In short, MAS, HLH, and related cytokine storm syndromes should be thought of as a spectrum of hyperinflammation, with the specific label varying with context (infection triggered, malignancy triggered, or autoimmune triggered). In the bottom line, they all share common pathways and benefit from prompt immunomodulation. Differentiating among them is less crucial than recognizing the syndrome and treating it early, while also addressing any underlying cause.

## 5. Overlaps with MIS-C and Pediatric Hyperinflammation

The COVID-19 pandemic revealed that viral infections can serve as potent triggers for HLH pathogenesis, with numerous patients developing secondary HLH/MAS, which worsened prognosis [25]. Specifically, MIS-C is a post-infectious hyperinflammatory condition associated with COVID-19 that has pathophysiologic overlap with MAS/HLH. Children with MIS-C often present with fever, shock or hypotension, elevated inflammatory markers (CRP, D-dimer, ferritin), and multi-organ involvement—features that can fulfill criteria for MAS. Indeed, MAS has been reported as a complication in MIS-C cases, contributing to more severe courses [16]. However, MIS-C is a distinct entity with specific diagnostic criteria (e.g., history of SARS-CoV-2 exposure, marked cardiac involvement like ventricular dysfunction or coronary aneurysms, etc.), so it is important not to mislabel every MIS-C case as HLH. The two syndromes can be concomitant: one analysis found that about 10–15% of MIS-C patients clearly exhibit MAS by expert review, and these children tended to be older and had more profound shock, cytopenias, and coagulopathy [16]. Because traditional HLH/MAS criteria (HLH-2004, MAS-2005, MAS-2016) were developed in other populations, they may not fully capture MIS-C-associated MAS. A 2024 study proposed preliminary, tailored criteria for MAS in MIS-C (Table 1): notably, ferritin > 469 μg/L and platelet count < 114×10^9^/L were identified as simple cutoffs that discriminated MIS-C with MAS (sensitivity 100%, specificity 94.9%) [16]. This low ferritin threshold (≈500) reflects that even moderate hyperferritinemia in MIS-C could indicate MAS on top of the baseline inflammation. Other signs like hypotension, splenomegaly, and CNS dysfunction were also more common in MIS-C patients with MAS [16]. The overlap of MIS-C and MAS has practical implications: both conditions respond to immunomodulatory therapy. In fact, standard MIS-C treatment already includes intravenous immunoglobulin (IVIG) and glucocorticoids, and in refractory cases, IL-1 blockade (anakinra) has been used, which is the same armamentarium used for HLH/MAS. Thus, clinicians managing MIS-C should be vigilant for signs of macrophage activation (e.g., dropping platelets with rising ferritin), since this may prompt escalation to high-dose steroids or second-line agents sooner [14,16]. From a research perspective, MIS-C provides a unique window into cytokine storm syndromes, as it shares features with classic HLH/MAS (e.g., hyperferritinemia, cytopenias) but also has differences (e.g., profound IL-17A and IL-10 elevations, frequent cardiac involvement). Ongoing studies are comparing the cytokine and proteomic profiles of MIS-C versus other hyperferritinemic syndromes [14], which may refine our diagnostic and therapeutic approaches. For now, MIS-C is best thought of as part of the hyperinflammation spectrum; when a child with MIS-C is critically ill or not improving, clinicians should strongly consider MAS and treat accordingly, rather than viewing MIS-C and HLH/MAS as mutually exclusive diagnoses.

### MAS in the Context of Zoonotic and Viral Infections

Beyond bacterial sepsis and MIS-C, zoonotic and viral pathogens can trigger MAS, including Crimean–Congo hemorrhagic fever, brucellosis, Q fever, rickettsioses, and influenza. Management balances urgent immunomodulation (e.g., corticosteroids, anakinra) with targeted antimicrobial/antiviral therapy and source control. Recognizing these associations—particularly in endemic regions—can expedite diagnosis and tailored care [25,26,27,28].

## 6. Therapeutic Approaches and Recent Advances

Despite the extensive research conducted over the past years on the host’s dysregulated immune response in sepsis, immunomodulating agents have failed to show clear therapeutic success. This lack of benefit is primarily due to marked heterogeneity of the clinical and immunological phenotypes of sepsis. Early characterization of precise immune dysregulation could enable personalized treatment strategies using adjunctive immunotherapeutic approaches [17]. Particularly managing MAS/HLH in septic shock, prompt immunomodulatory therapy is the cornerstone, alongside optimal supportive care and treatment of the inciting cause. Given the life-threatening nature of HLH/MAS, therapies are often initiated empirically (based on high ferritin and clinical suspicion) without waiting for complete diagnostic confirmation [4]. Key elements of management include the following (Table 2):

(i) Concurrent Infection Control: In sepsis-associated HLH, appropriate antibiotics or antivirals must be administered to address the trigger, even if immunosuppressive therapy is started [4]. The 2024 consensus is that controlling the known or suspected trigger (e.g., broad-spectrum antibiotics for bacterial sepsis) should happen in parallel with HLH treatment, not in sequence [47]. Similarly, removal of potential triggers (draining abscesses, stopping culprit drugs, or treating malignancy if present) is crucial.
(ii) Supportive Care: Patients often require ICU support for organ dysfunction (vasoactive support in shock, ventilation for acute respiratory distress syndrome (ARDS), dialysis for acute kidney impairment (AKI)). Coagulopathy and cytopenias in MAS may necessitate blood product transfusions [43]. Aggressive supportive care cannot replace immunotherapy in HLH, but it can buy crucial time for it to take effect. Prognostic discussions should reflect that early immunomodulation has the potential to dramatically reverse even severe organ failure.
(iii) Corticosteroids: High-dose corticosteroids remain the backbone therapy for both pediatric and adult HLH/MAS [13]. Steroids have broad anti-inflammatory effects and speed the suppression of cytokine production. Typical regimens include IV methylprednisolone (for example, 1–2 mg/kg up to pulse doses ~30 mg/kg/day for 3–5 days in severe cases) [5]. In critically ill patients, steroids are often started at diagnosis; if HLH is confirmed, a dexamethasone-based regimen (as per HLH-94 protocol) may be continued. Rapid improvement in fever spikes, ferritin levels, and clinical status often follows steroid initiation if HLH/MAS is the correct diagnosis.
(iv) Intravenous Immunoglobulin (IVIG): IVIG is commonly given in pediatric hyperinflammatory syndromes, such as Kawasaki disease and MIS-C, and has been used in infection-associated HLH as well. IVIG can help neutralize pathogens and provide immune modulation. Some protocols incorporate IVIG 1–2 g/kg, especially when an underlying infection like EBV is suspected, or in MAS complicating Kawasaki disease or sepsis [14]. However, IVIG is not recommended as a standard treatment in sepsis treatment guidelines due to its lack of therapeutic benefit [33]. More research is needed on this field in septic patiets with MALS. While IVIG alone is usually insufficient to control full-blown HLH, it may serve as a useful adjunct in milder cases or as a temporary bridge in resource-limited settings.
(v) Etoposide-Based Therapy: Etoposide, a chemotherapeutic agent, is a main component of the standard HLH protocol (HLH-94 and HLH-2004), as it induces apoptosis of overactive immune cells (particularly T cells). In patients with established HLH (especially familial or malignancy-associated HLH), etoposide + dexamethasone therapy significantly improves survival and is considered definitive therapy [35,47]. However, in sepsis-associated HLH, the decision to use etoposide is nuanced due to concerns about severe myelosuppression and infection risk in an already septic patient. Recent practice has been trending towards using biologic immunomodulators (like anakinra) first and reserving etoposide for refractory cases [4]. Nonetheless, if a patient fails to respond to first-line therapy or if genetic HLH is strongly suspected, experts will initiate etoposide even in adults. The 2024 HLH consensus guidelines affirm that HLH-94 therapy can be life-saving in adults with secondary HLH, despite historically being a pediatric regimen [35]. Thus, etoposide remains in the arsenal, but newer therapies have thankfully reduced the frequency with which it is needed in sepsis/MAS scenarios.
(vi) Cyclosporine A (CSA): CSA, a calcineurin inhibitor, is another conventional HLH/MAS treatment that suppresses T-cell activation. In rheumatology, CSA is often combined with steroids as first-line for MAS in sJIA. In infection-associated HLH, CSA can be added early, especially if there is only a partial steroid response. However, practice is shifting—some centers favor IL-1 blockade (anakinra) instead of CSA up front, due to CSA’s renal/hepatic toxicities and slower onset [48]. For example, many institutions in the United States now start anakinra with high-dose steroids as initial therapy and reserve cyclosporine for later [4]. CSA may still be used as an adjunct if needed (e.g., ongoing MAS features after anakinra and steroids), at doses ~2–7 mg/kg/day, aiming for therapeutic trough levels.
(vii) IL-1 Blockade (Anakinra): Interleukin-1 is a key pro-inflammatory cytokine in MAS, and anakinra (recombinant IL-1 receptor antagonist) has emerged as a front-line therapy for hyperferritinemic syndromes. Anakinra has the advantages of a quick onset, short half-life, and a favorable safety profile even in infection. It blocks IL-1-mediated inflammation without broadly suppressing adaptive immunity [49]. A pivotal post hoc analysis in adults with septic shock and secondary HLH features showed that adding anakinra reduced mortality, especially in those with DIC and hepatobiliary dysfunction [12]. Moreover, the PROVIDE trial demonstrated that, based on specific immune phenotypes along with other parameters in septic adults, anakinra treatment led to improved clinical outcomes [50]. In pediatric MAS, case series have reported rapid remission of hyperinflammation with anakinra, including refractory cases where steroids and CSA had failed [4,18,39]. By 2024, consensus recommendations frequently list anakinra as the preferred second-line agent for HLH/MAS if there is an inadequate response to steroids [49]. Many experts will initiate anakinra concurrently with steroids in fulminant cases, given the high lethality of MAS [51]. Dosing is higher than typical rheumatoid dosing—often 4–8 mg/kg/day, divided 2–4 times daily or given as a continuous infusion in critically ill patients [49]. High-dose anakinra has been used safely in septic shock trials (even up to 48 mg/kg/day IV) [49]. The only real drawbacks are logistical (need for repeated dosing or infusion) and cost. Overall, anakinra has become a pillar of therapy, bridging the gap between traditional immunosuppressants and targeted biologics. Early use of anakinra in sepsis-associated HLH can halt the progression of the cytokine storm and has been associated with recovery of organ function in many reports [12].
(viii) IL-6 Inhibition: Tocilizumab (anti-IL-6 receptor) is well-known for treating cytokine storms in CAR-T cell therapy and severe COVID-19. IL-6 is often markedly elevated in MAS; although IL-1 blockade is usually favored, tocilizumab has been used in refractory MAS. Case reports suggest it can be effective when ferritin remains high despite anakinra [41,42]. Caution is warranted, as IL-6 inhibition can mask fever and cause paradoxical increases in serum ferritin (making clinical monitoring tricky). Currently, evidence from clinical trials remains insufficient to support the use of tocilizumab in sepsis treatment.
(ix) Interferon-γ Neutralization: Emapalumab, a monoclonal antibody against IFN-γ, was approved for primary pediatric HLH and has shown activity in secondary HLH as well. IFN-γ is a central driver of macrophage activation, so its blockade can dampen the HLH cascade [44]. Due to limited availability and cost, emapalumab is generally reserved for refractory cases or considered in known familial HLH [44].
(x) JAK Inhibitors: Ruxolitinib, a JAK1/2 inhibitor, can broadly suppress the cytokine circuit by interfering with signaling of multiple interleukins and interferons. Emerging evidence (case series and phase II trials) indicates that ruxolitinib can salvage patients with refractory HLH [45]. It has been incorporated into some clinical protocols (e.g., the HLH-94 “RUXO” experimental arm) [45] and is being studied in combination with steroids in secondary HLH. As of 2025, ruxolitinib is not yet standard first-line therapy but is a promising option for severe cases not responding to IL-1/IL-6 blockers and steroids.
(xi) Other Targets: In specific scenarios, additional therapies are considered. For Epstein–Barr virus-driven HLH (often seen in teens/adults), rituximab (anti-CD20 monoclonal) can help clear EBV-infected B cells and has been added to HLH regimens [4]. Intravenous etoposide, as noted, remains a key option for cytotoxic debulking of the immune response. Experimental approaches like anti-IL-18 (tadekinig alfa) are under investigation given the extraordinarily high IL-18 levels in some MAS patients [10]. Lastly, extracorporeal blood purification therapies are a group of treatments that may modulate the host’s inflammatory response by removing inflammatory mediators and/or circulating bacterial toxins. However, significant practical challenges remain and require consideration. Plasmapheresis has been used in hyperferritinemic sepsis to remove inflammatory mediators—some centers report stabilization in otherwise refractory cases, though evidence is not yet definitive [48].

**Figure 2:**
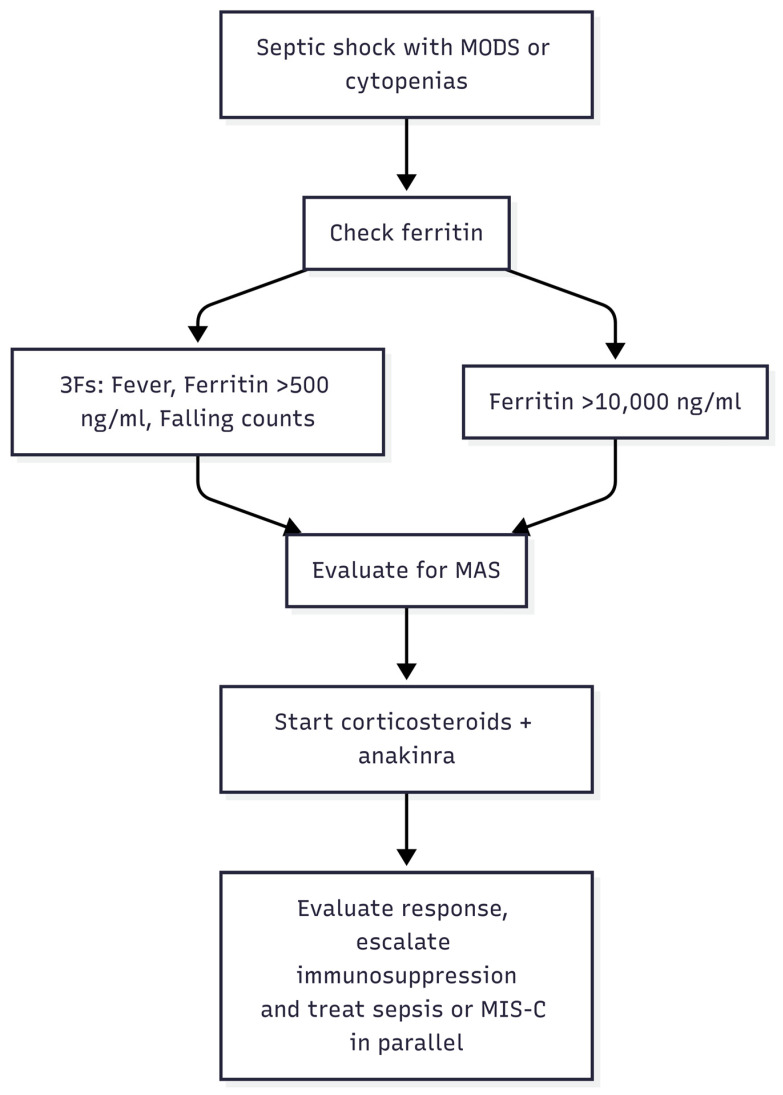
Recognition and Treatment Algorithm for MAS in Septic Shock. MODS, multiorgan dysfunction syndrome; MAS, macrophage activation syndrome; MIS-C, multisystem inflammatory syndrome in children.

**Clinical Course and Outcomes:** With these therapies, survival in HLH/MAS has improved markedly over the past two decades. Where historically HLH was 95% fatal, recent multicenter experiences report survival rates on the order of 60–80% even in critical illness. Nonetheless, in the septic shock population, MAS confers significant mortality risk. As mentioned, pediatric septic shock with MAS had ~4-fold increased mortality [12], and adult septic shock patients meeting HLH criteria have reported death rates of 50% or higher despite ICU care. Early recognition and treatment are therefore vital. In 2024, an international consensus (HiHASC Collaboration) published guidelines stressing a systematic approach to recognize HLH/MAS early, using the 3-F mnemonic and urgent diagnostic evaluation, and to institute therapy promptly even while awaiting confirmatory tests [23] (Table 3). Very recent ongoing trials are testing some of the above biologics (e.g., anakinra, emapalumab, ruxolitinib) in controlled settings for sepsis-associated hyperinflammation [52]. The hope is that by tamping down the “friendly fire” of the immune system in septic shock, we can improve outcomes without compromising infection control. The current paradigm is multi-disciplinary management involving intensivists, hematologists, rheumatologists, and infectious disease specialists to tailor treatment for each patient. Every septic shock patient with unexplained cytopenias, organ failure, and high ferritin represents a race against time to diagnose MAS/HLH. The latest literature reinforces that vigilance and early immunomodulation save lives in this hyperferritinemic subset of sepsis [12,38,53] (Figure 2).

Table 4. Sepsis-adapted prompts and diagnostic pathway (HiHASC 2023 ‘3-F’ mnemonic—fever, falling blood counts, ferritin), with suggested escalation steps for pediatric septic shock complicated by MAS (Cox et al., 2024 [23]).

## 7. Conclusions and Future Directions

Hyperinflammatory syndromes in children are life-threatening manifestations of immune dysregulation, and their overlap with severe infections, autoimmune flares, and MIS-C continues to obscure timely recognition. Despite increasing awareness, progress in diagnosis and treatment has been fragmented, and most tools remain extrapolated from adult or oncology-based HLH cohorts.

Future priorities must include the development of pediatric-specific, sepsis-adapted diagnostic criteria, informed by ferritin kinetics and integrated with cytokine and immunophenotyping signatures. Equally urgent is the design of biomarker-driven, prospective clinical trials to define when and how to use corticosteroids, biologics, and emerging targeted agents in children.

To translate these advances into survival benefit, the field must adopt risk-stratification algorithms and protocolized treatment pathways, tested in real-world pediatric critical care settings and disseminated globally. Education of pediatric intensivists and frontline teams is critical to ensure early recognition and uniform care.

The next phase requires a shift from merely recognizing MAS to delivering precision immunotherapy within standardized sepsis care protocols—a step that can transform outcomes for critically ill children worldwide. Achieving this transition—from descriptive recognition to precision, protocolized immunotherapy—represents the most important opportunity in decades to change the natural history of hyperinflammatory shock in children.

## Figures and Tables

**Table 2:** Pediatric-specific summary of MAS in septic shock.

Category	Category/Tool	Pediatric-Specific Points	Type of Studies	Population	Key References
**Diagnostics**	Hyperferritinemia thresholds	Ferritin > 500–700 ng/mL identifies hyperferritinemic sepsis, with markedly higher mortality and MAS features. Dynamic rise with cytopenias and hypofibrinogenemia strengthens suspicion. Ferritin ≥ 1000–2000 ng/mL signals higher risk; values ≥ 10,000 ng/mL are highly specific for HLH but not exclusive.	Multicenter pediatric cohorts; reviews	Pediatric only	Fan et al., 2023 [12]; Valerie et al., 2023 [5]; Pai et al., 2025 [20]; Demirkol et al., 2012 [29]
**Diagnostics**	HLH-2004 diagnostic criteria	5/8 features (fever, splenomegaly, cytopenias, hypertriglyceridemia and/or hypofibrinogenemia, hemophagocytosis, ferritin ≥ 500 µg/L, low NK-cell activity, high sCD25). In septic shock, criteria overlap; performance limited. Bone marrow hemophagocytosis is neither necessary nor sufficient.	Guideline consensus; widely used standard	Pediatric; not sepsis-specific	Henter et al., 2007 [6]; Knaak et al., 2020 [22]; Bursa et al., 2021 [30]
**Diagnostics**	2016 EULAR/ACR/PRINTO MAS (sJIA) criteria	Ferritin ≥ 684 ng/mL plus ≥ 2 of: platelets ≤ 181 × 10^9^/L, AST > 48 U/L, triglycerides ≥ 156 mg/dL, fibrinogen ≤ 360 mg/dL. Robust in sJIA; not validated in de-novo sepsis, but overlap with septic MAS phenotypes observed.	expert consensus + validation cohorts	Pediatric rheumatology	Ravelli et al., 2016 [7]
**Diagnostics**	HScore	Scoring system originally adult; incorporates cytopenias, hepatosplenomegaly, fever, hypertriglyceridemia, elevated AST, low fibrinogen, hemophagocytosis, immunosuppression. Pediatric data (cutoff ≥ 169) show high sensitivity (~100%) and specificity (~80%) for HLH, but not validated in sepsis.	expert consensus. Comparative study	Mixed; Adult derivation; retrospective pediatric validation	Fardet et al., 2014 [15]; Knaak et al., 2020 [22]; Canna and Marsh 2020 [31]; Chinnici et al., 2023 [32]
**Diagnostics**	Biomarkers (sCD25, CXCL9/IL-18, sCD163)	High sCD25 (>10,000 U/mL) supports HLH; CXCL9 tracks IFN-γ activation; sCD163 elevated in MAS. May help distinguish hyperinflammatory endotypes. Limited sepsis-specific pediatric validation.	Small pediatric cohorts; translational studies	Pediatric (partial adult extrapolation)	Tang et al., 2021 [9]; Nguyen et al., 2024 [13]; Canna and Marsh 2020 [31]; Chinnici et al., 2023 [32]
**Treatment**	High-dose corticosteroids (methylprednisolone)	First-line for suspected MAS once antimicrobials initiated. Typical dosing: 1–2 mg/kg/day to pulse 30 mg/kg/day (max 1 g) in fulminant cases. Pediatric SSC 2020 recommends hydrocortisone only in catecholamine-refractory shock, not routine use.	Case series; expert consensus; SSC guideline	Pediatric	Weiss et al., 2020 [33]; Lee et al., 2024 [4]
**Treatment**	Intravenous immunoglobulin (IVIG)	⚠ Not recommended for routine MAS in sepsis. Sometimes used early in MAS/MIS-C overlap or selected toxin-mediated syndromes.	Guidelines (against in generic sepsis); observational	Mixed; not MAS-specific	Inguscio et al., 2025 [14]; Weiss et al., 2020 [33]
**Treatment**	Etoposide-based protocols (HLH-94/2004) [6]	Reserved for proven HLH or refractory MAS with organ failure after steroids/biologics. Evidence stronger in pHLH and EBV-HLH; high toxicity risk in sepsis.	Prospective pediatric (pHLH); retrospective secondary HLH; adult analyses	Pediatric + adult extrapolation	Bergsten et al., 2017 [34]; La Rosée et al., 2019 [35]; Naymagon et al., 2025 [36]; Böhm et al., 2024 [37]
**Treatment**	Cyclosporine A (CSA)	Second-line for steroid-refractory MAS; 2–7 mg/kg/day. Rapid clinical response in rheumatology MAS; less evidence in infection-triggered cases.	Pediatric case series; consensus	Pediatric	Clinical analysis of MAS in pediatric autoimmune disease [38]; Lee et al., 2024 [4]
**Treatment**	IL-1 blockade (Anakinra)	Expanding pediatric use in MAS and hyperferritinemic MODS. Doses of 4 up to 48 mg/kg/day IV in sepsis; no pediatric RCT in sepsis; adult sepsis re-analysis suggests benefit in MAS-like endotype.	Pediatric case series; adult RCT subgroup; ongoing pediatric RCT	Pediatric + adult extrapolation	Rajasekaran et al., 2014 [18]; Shakoory et al., 2016 [39]; Hall et al. TRIPS trial 2025 [40]; Silencing Cytokine Storm review 2025 [35]
**Treatment**	anti-IL-6 receptor (Tocilizumab)	Well-known therapeutic agent for treating cytokine storms in CAR-T cell therapy and severe COVID-19. Promising in managing paediatric septic shock. Evidence from clinical trials remains insufficient to support the use of tocilizumab in sepsis treatment.	Case reports and small paediatric case series	Mixed	Paranga et al., 2024 [41]; Majidpoor et al., 2022 [42]
**Treatment**	IFN-γ blockade (Emapalumab)	Approved for refractory primary HLH. Pediatric case reports/series describe use in MAS, including infection-triggered cases. No septic shock trial data.	Case reports/series; regulatory approval	Pediatric; extrapolated indication	[43,44]
**Treatment**	JAK inhibition (Ruxolitinib)	Emerging salvage option in refractory HLH/MAS; pediatric retrospective series show feasibility, especially EBV-driven. No septic-shock trials.	Early pediatric retrospective cohorts	Pediatric (minimal adult extrapolation)	[45,46]

**Table 3:** Therapeutic Options for HLH/MAS in Septic Shock.

Therapy	Mechanism	Typical Use	Comments	Key References
Corticosteroids	Broad anti-inflammatory; cytokine suppression	First-line for HLH/MAS	High-dose IV methylprednisolone or dexamethasone	Lee et al., 2024 [4]; Weiss et al., 2020 [33]; Bergsten et al., 2017 [34]
IVIG	Immunomodulation; pathogen neutralization	Adjunct in MAS, Kawasaki, MIS-C	May be used in milder cases or as a bridge	Inguscio et al., 2025 [14]; Weiss et al., 2020 [33]
Etoposide	Cytotoxic to activated T cells	Definitive therapy in HLH	High toxicity; reserved for severe/familial/refractory cases	Bergsten et al., 2017 [34]; La Rosée et al., 2019 [35]; Böhm et al., 2024 [37]
Cyclosporine A	Calcineurin inhibitor; T-cell suppression	Add-on in MAS/HLH with partial steroid response	Nephrotoxic, slower onset; often replaced by biologics	Clinical analysis of MAS in pediatric autoimmune disease [38]; Lee et al., 2024 [4]
Anakinra	IL-1 receptor antagonist	Second-line or adjunct; increasingly first-line	Rapid onset; favorable safety in sepsis; IV/SC options	Rajasekaran et al., 2014 [18]; Shakoory et al., 2016 [39]; Hall et al., 2025 [40]
Tocilizumab	IL-6 receptor blockade	Refractory MAS; COVID-19 cytokine storm	Can mask fever; raises ferritin paradoxically	Paranga et al., 2024 [41]; Majidpoor et al., 2022 [42]
Emapalumab	IFN-γ neutralization	Approved for primary HLH; off-label in secondary	Expensive; limited availability; used in refractory cases	Garonzi et al., 2021 [44]; Slaney et al., 2023 [43]
Ruxolitinib	JAK1/2 inhibitor—broad cytokine suppression	Refractory HLH/MAS	Experimental but promising in trials	Huarte et al., 2021 [45]; Guo et al., 2025 [46]
Rituximab	CD20+ B-cell depletion	EBV-driven HLH	Used in combination with HLH-94 protocols	Wu et al., 2024 [47]
Plasmapheresis	Removes inflammatory mediators	Adjunct in hyperferritinemic sepsis	Limited evidence; salvage therapy	Shakoory et al., 2016 [39]

**Table 4:** Sepsis-adapted prompts and diagnostic pathway (HiHASC 2023 ‘3-F’ mnemonic).

Step	Prompt	Details	Key References
Initial suspicion	3-F mnemonic	Fever, falling blood counts, ferritin (hyperferritinemia > 500–1000 ng/mL)	Cox et al., 2024 [23]
Diagnostic workup	Laboratory + clinical evaluation	Apply HLH-2004/MAS-2016 criteria, HScore; assess cytopenias, fibrinogen, soluble IL-2R, NK-cell function if available	Henter et al., 2007 [6]; Ravelli et al., 2016 [7]; Fardet et al., 2014 [15]; Knaak et al., 2020 [22]
Therapeutic escalation	If MAS/HLH strongly suspected	Initiate corticosteroids ± anakinra early; continue antimicrobial coverage in parallel	Lee et al., 2024 [4]; Rajasekaran et al., 2014 [18]; Shakoory et al., 2016 [39]
Advanced therapy	Refractory or severe cases	Consider cyclosporine, etoposide, biologics (tocilizumab, emapalumab, ruxolitinib) with multidisciplinary input	La Rosée et al., 2019 [35]; Böhm et al., 2024 [37]; Garonzi et al., 2021 [44]
Supportive care	ICU-level organ support	Shock management, ventilation, dialysis, transfusions as needed; early consultation with hematology/rheumatology	Weiss et al., 2020 [33]; Carcillo et al., 2019 [11]

## Data Availability

No new data were created or analyzed in this study. Data sharing is not applicable to this article.

## Abbreviations

MAS	Macrophage Activation Syndrome
HLH	Hemophagocytic Lymphohistiocytosis
MIS-C	Multisystem Inflammatory Syndrome in Children
MALS	Macrophage Activation-Like Syndrome
DIC	Disseminated Intravascular Coagulation
IVIG	Intravenous Immune Globulin
MODS	Multi-Organ Dysfunction Syndrome
CSA	Cyclosporine A
NK	Natural Killer
CRS	Cytokine Release Syndrome
CSS	Cytokine Storm Syndrome
SARS-CoV-2	Severe Acute Respiratory Syndrome Coronavirus 2
IV	Intravenous
IL-1	Interleukin 1
IL-6	Interleukin 6
IFN-γ	Interferon Gamma
